# Technological considerations for genome-guided diagnosis and management of cancer

**DOI:** 10.1186/s13073-016-0370-4

**Published:** 2016-10-26

**Authors:** Niall J. Lennon, Viktor A. Adalsteinsson, Stacey B. Gabriel

**Affiliations:** Broad Institute of MIT & Harvard, Cambridge, MA 02142 USA

## Abstract

Technological, methodological, and analytical advances continue to improve the resolution of our view into the cancer genome, even as we discover ways to carry out analyses at greater distances from the primary tumor sites. These advances are finally making the integration of cancer genomic profiling into clinical practice feasible. Formalin fixation and paraffin embedding, which has long been the default pathological biopsy medium, is now being supplemented with liquid biopsy as a means to profile the cancer genomes of patients. At each stage of the genomic data generation process—sample collection, preservation, storage, extraction, library construction, sequencing, and variant calling—there are variables that impact the sensitivity and specificity of the analytical result and the clinical utility of the test. These variables include sample degradation, low yields of nucleic acid, and low variant allele fractions (proportions of assayed molecules carrying variant allele(s)). We review here the most common pre-analytical and analytical factors relating to routine cancer patient genome profiling, some solutions to common challenges, and the major sample preparation and sequencing technology choices available today.

## Background

Technologies that profile the cancer genome are powerful tools to elucidate molecular mechanisms that contribute to the pathogenesis, progression, regression, and resistance of neoplastic disease [1]. Over the past 5 years, our understanding of these mechanisms has improved, in part due to projects such as The Cancer Genome Atlas (TCGA) [2]. Accordingly, applications for tumor molecular profiling have become increasingly translational. Genomic testing of patient tumors is now used in diagnostics [3], precision therapy selection [4], disease progression monitoring (mostly in a clinical research setting) [5], and clinical trial enrolment [6]. However, mapping the cancer genome is not a simple task. Each individual’s cancer genome contains a multitude of alterations and alteration types (for example, single base changes, structural variation, epigenetic changes) that require specific wet lab and analytical approaches for optimal performance of genomic profiling.

Profiling the cancer genome of a patient sample is complex and fraught with opportunities for technical artifacts, reduced sensitivity, false-positive findings, and outright test failure. Annotation, interpretation, and reporting of clinically relevant variants encompass the process by which genomic data are translated into the practice of medicine. At each of the steps to produce genomic data—sample collection, nucleic acid extraction, library preparation, sequencing, and variant calling—one must consider how technical and methodological decisions might impact the sensitivity and specificity of the data that will be delivered to a clinician for the provision of patient care. We present here a review of the major technical considerations, test selection considerations, sequencing technologies, and analytical variables that impact cancer genomics.

## Pre-analytical considerations

**Fig. 1:**
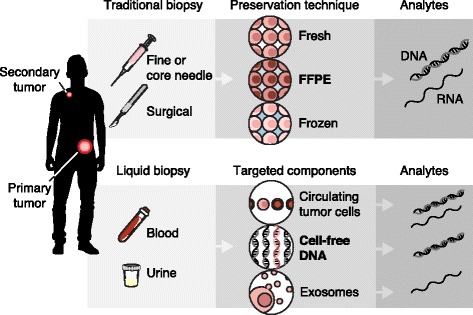
Overview of the most commonly used biopsy techniques, preservation methods, and genomic analytes. Traditional biopsy methods include fine- or core-needle biopsy or surgical resection. These biopsies typically only access the primary tumor site. From traditional tissue biopsy the most common pathological preservation path is through formalin fixation and paraffin embedding (FFPE), though fresh frozen tissue or disaggregated cells are sometimes also available. From each of these material types, both DNA and RNA can be extracted. Liquid biopsy usually involves blood draw, though some groups are now testing urine and other body fluids. Liquid biopsy can have representative somatic lesions from more than one tumor site. Circulating tumor cells (CTCs), cell-free DNA (cfDNA), and exosomes or extracellular vesicles (EVs) are the most common components of liquid biopsy that are isolated for somatic analysis. DNA and RNA can be isolated from CTCs, but only DNA is represented in the cfDNA extraction, and RNA is most commonly targeted from EVs

Sample collection, preservation, and manipulation are important pre-analytical factors to consider prior to genomic data generation (Fig. 1). Traditional methods for tumor biopsy include fine- or core-needle aspiration or surgical resection. Formalin fixation and paraffin embedding (FFPE) is most often used for sample preservation though fresh frozen tissue or disaggregated cells are sometimes used for specific downstream applications. Recently, liquid biopsy has emerged as a potentially powerful and minimally invasive alternative for routine monitoring and characterization of cancer. Here we describe the most common sampling methods and their relative advantages and disadvantages for genomic profiling.

### Formalin fixation and paraffin embedding

For a long time, FFPE has been used to preserve and solidify tumor biopsies for morphological examination [7]. While visually examining patient slides under the microscope, pathologists of the early 20th century could hardly have imagined the additional information locked inside the immobilized tissue sections before them. Fast-forward to today, the methods for fixation might not have changed much, but the methods for extracting and utilizing molecular information about a patient’s cancer have advanced to the point of clinical significance.

FFPE has proven utility for morphological and immunohistochemical interrogation of cancerous cells; however, the use of FFPE poses several challenges to molecular characterization of genomic material [4]. Cell pellets and fresh frozen tissue routinely yield >10 μg DNA; however, in our experience with several thousand FFPE samples (as blocks, slides, or scrolls), they generally yield ≤1 μg DNA (unpublished data). Depending on the intended use of the genomic material, the amount of DNA yielded from FFPE samples might not be sufficient to produce high complexity sequencing libraries, which limits the sensitivity of variant calling. In addition to yield, the quality of extracted material can vary widely due to the interaction of formaldehyde with DNA. Several studies have reported both decreased yield and quality (measured by integrity and fragment length of extracted DNA) of FFPE-derived DNA with increasing length of storage [8, 9], though our experience is that even recently fixed samples can vary in quality across different submitting labs, suggesting that variation in processing protocols or reagents is a factor (unpublished data). Even seemingly good quality DNA extracted from FFPE samples can have higher variant false-positive rates compared to DNA from non-FFPE samples due to artifactual base changes resulting from formalin cross-linking of cytosine nucleotides [10].

In response to these issues, several methods have been developed to assess quality and quantity of extracted DNA (for example, using quantitative PCR to measure ratios of amplicons of increasing lengths), which can help to better triage incoming samples and, where appropriate, modify laboratory protocols (for example, by pooling of samples with similar quality scores together or using DNA repair enzymes prior to library construction) to maximize data utility [11–13]. Similarly, several sample preparation techniques have been developed to specifically process FFPE-derived (or otherwise degraded or low yielding) DNA samples, including some that leverage both DNA repair steps and alternative, more efficient adapter ligation strategies [14], while others have optimized automated library construction methods that use high-efficiency enzymes and have produced acceptable results for many FFPE samples [4]. Furthermore, downstream variant-calling pipelines can detect some of the more common artifactual base changes through filtering [15], which highlights the need to capture and propagate sample type information to the analytical pipeline for optimal performance.

Generation of high quality genome sequencing data from FFPE-derived RNA is considerably more challenging than from FFPE-derived DNA. RNA extraction yields are generally higher than those of DNA (>10 μg; unpublished data), but FFPE-derived RNA is often highly degraded. Recently, methods for quality control of FFPE-derived RNA have been reported [16] and targeted selection methods have demonstrated utility in the generation of data to analyze transcriptomes and druggable fusions [17, 18].

As molecular profiling becomes more routine in clinical management, it remains to be seen if non-crosslinking tissue preservatives (for example, Optimal Cutting Temperature compound (OCT); PAXgene) might be used more frequently, considering that the improved quality of extracted nucleic acids can come at the expense of immunohistochemical performance [19].

### Fresh frozen tissue and cells

Many of the integrity and yield issues associated with FFPE-derived material are avoided by the use of fresh frozen tissues and bulk cell pellets. Nonetheless, artifacts can still be introduced in the sample preparation process that are exacerbated by contaminating reactive elements in extraction buffers. Notably, high-energy acoustic shearing can mediate transversion artifacts through nucleic acid oxidation, which appear at low allele fractions [20]. This highlights how care must be taken at each step in the sequencing process, from nucleic acid extraction to sample preparation and detection, to avoid introduction of artifacts and biases that ultimately impact the sensitivity and specificity of clinical tests.

A specialized set of procedures is required to capture and sequence single cells. A common pre-analytical pipeline for single cell isolation is to disaggregate fresh tumor biopsy material followed by fluorescence activated cell sorting (FACS) prior to library preparation [21]. More efficient methods, such as micromanipulation (for example, circulating tumor cell enrichment and isolation from blood [22]), might be required for isolation of rare cells. Microfluidic isolation based on cell size has also been described [23].

### Liquid biopsy

Genomic profiling from liquid biopsy is a rapidly growing area due to the relative ease of collection and lower associated costs. The total cost to obtain a surgical biopsy ranges from approximately $1000–4000 [24], whereas to obtain and extract nucleic acids from a liquid biopsy costs $100–200. Additionally, while tumor biopsy is the standard of care for primary diagnosis, tissue biopsies are not generally taken to monitor disease progression or to test metastatic lesions.

Multiple forms of liquid biopsy, such as cell-free DNA (cfDNA) [25], circulating tumor cells (CTCs) [26], and extracellular vesicles (EVs) [27], can be isolated from blood among other bodily fluids (see Fig. 1). Key considerations for molecular profiling of genetic information from lipid biopsies include special requirements for sample processing, low yield and purity of tumor-derived nucleic acids, and the uncertain false-negative rate.

Liquid biopsies are particularly sensitive to how they are handled, up to a certain point. For instance, blood must be properly collected (for example, into specialized blood collection tubes to minimize cellular DNA release [28]), stabilized, and fractionated within hours to days to mitigate degradation of cells or nucleic acids [29, 30]. Plasma fractionated from blood can be frozen for extraction of cfDNA or nucleic acids from EVs at a later date. For analysis of CTCs, positive selection (isolation of a target cell population by using an antibody that specifically binds that population) or negative depletion (depletion of all cell types except the cell type of interest) must be performed on the buffy coat (the fraction of an anticoagulated blood sample that contains most of the white blood cells and platelets following density gradient centrifugation) or whole blood prior to freezing an enriched cell pellet [31] (or single CTCs, if further purified [22]).

Liquid biopsies usually yield picogram to nanogram quantities of DNA or RNA, of which only a small fraction is derived from tumors [32]. In most individuals, peripheral blood mononuclear cells (PBMCs) and other non-tumor cells constitute the predominant source of cfDNA in blood [33]; similarly, methods to enrich for CTCs often result in significant carryover of PBMCs. In cancer patients, tumor purity in extracted cfDNA or enriched CTC samples is usually <5 % [32] and it is challenging to quantify tumor-derived EVs [22, 27]. If the total yield of nucleic acids is too low, whole genome amplification (WGA) or whole transcriptome amplification (WTA) might be required but can distort the original template [34]. Furthermore, the sensitivity to detect variants from low purity samples will be limited by the total yield or genome equivalents of cfDNA that are available for sequencing. Thus, the accurate profiling of tumor DNA or RNA in a sample that contains non-tumor DNA or RNA is challenging and requires specialized methods, such as error-correcting with molecular barcodes (tags of parsable (separable by software) sequence that are used to label individual starting molecules), also known as unique molecular indexes (UMI) [35], high efficiency library preparation kits for low input material [36, 37], or mutation enrichment [38]).

The false-negative rate in liquid biopsies is often difficult to determine. Tumor-derived cfDNA, EVs, or CTCs are sometimes undetectable in blood owing to technical or biological reasons. CTCs are not always enumerated prior to sequencing and might vary in quality of nucleic acids (for example, from apoptotic cells [39]) or might not express the surface markers used for identification. Similarly, detection methods for tumor-derived cfDNA or EVs often require probing for a select set of alterations and might not always include those present in a patient’s cancer. Nucleosome positioning might also have an effect on the false-negative rate of sequencing cfDNA [33]. For these reasons, a negative result in a liquid biopsy assay might warrant follow-up testing from a tissue biopsy. Table 1 provides a summary of common pre-analytical issues, impacts, and contingencies associated with different sample types.

**Table 1:** Common pre-analytical and sample preparation issues related to different sample types

Sample type	Common issues	Impact	Contingencies/solutions
Formalin-fixed, paraffin-embedded (FFPE)	• Low yield of DNA • DNA degradation • DNA base modification • RNA degradation	• Reduced complexity libraries; library failure; decreased sensitivity • Reduced complexity; library failure; decreased sensitivity • Increased false positive rate • Library failure; high duplication	• DNA repair; pooling of indexed libraries prior to capture (exomes or panels); specialized low input library methods • DNA repair; short amplicon amplification; specialized library methods • FFPE-aware filtering of variants; DNA repair • Selection-based or targeted preparation instead of polyA-based preparation
Fresh frozen tissue of bulk cells	• Buffer or process-induced modification of DNA bases	• Increased false positive rate	• Chelation of oxidative species; oxidation aware filtering
Single cells	• Low DNA yield • Whole genome amplification (WGA) bias • Low RNA yield	• Library failure • Increased false positives and false negatives • Library failure	• WGA • Optimized WGA • Whole transcriptome amplification (WTA)
Liquid biopsy	• Low DNA yield of cfDNA • Low purity of ctDNA in cfDNA • Low DNA yield from CTCs • Low RNA yield and quality from CTCs • Low RNA yield from EVs	• Library failure; reduced sensitivity • Reduced sensitivity • Library failure; reduced sensitivity; reduced specificity • Library failure • Library failure	• Optimized library preparation; specialized library preparation • High sequencing depth; molecular barcoding (UMIs) • WGA • WTA; specialized library preparation. • WTA; specialized library preparation.

## Matching the test to the intended use

Reduced costs in the generation of massively parallel sequence data and advances in wet lab and analytical techniques have resulted in a wide variety of options for tumor molecular profiling. Whole genome sequencing (WGS) [40], whole exome sequencing (WES) [4], large (300–600 gene) panels [3, 41, 42], small (<50 genes) panels [43], and hotspots (specific mutations in somatic genes) [44] have been used for somatic alteration profiling (Table 2). Selection of a specific genomic profiling test requires consideration of both pre-analytical (sample source) and analytical factors. One very important factor to consider is the intended use of the test.

**Table 2:** Common sequencing-based tests used in cancer genomics: their targeted regions, primary use cases, and limitations

Sequencing assay	Targeted regions	Primary use	Limitations
Whole genome sequencing	All genes, all exons, all non-coding regions	Discovery	Cost; depth; limited sensitivity for low allele fraction
Whole exome sequencing	All genes, all exons	Clinical research; panel-negative diagnostic testing; neo-epitope prediction	Cost; depth; moderate sensitivity for low allele fraction
Large gene panel	300–600 genes	Diagnostics; clinical trials; clinical research	Breadth; neo-epitope prediction
Small gene panel	<100 genes	Diagnostics; disease progression monitoring	Breadth; neo-epitope prediction
Hotspot panel	Portions of 50–80 genes, specific exons, variants	Diagnostics	Breadth; neo-epitope prediction
Transcriptome	mRNA	Variant validation; neo-epitope expression; fusion calling	Cost
Targeted RNA panel	Fusion genes	Fusion calling	Breadth; variant validation capability limited to targeted territory

Somatic variant calling from tumor genomic data is a complex and highly context-specific activity. Generally, variant sensitivity is a function of the depth of unique, high quality sequence reads at a site (read depth) and the proportion of molecules in the sample that are derived from the cancerous cells, known as the tumor allele fraction (AF) [45]. Tumor allele fraction is impacted by purity of the biopsy material, that is, how much “contamination” of normal DNA exists from non-cancer cells, and by the heterogeneity of the cancer itself. Tests that seek to assay known cancer driver genes or hotspots typically aim for high sensitivity to call these specific variants and are less concerned with novel or false positive incidental events. To achieve acceptable sensitivity (>99 %) for clinical use in solid tumor fresh frozen or FFPE samples, tests are typically run on samples with >20 % tumor purity (AF) and to high-read depths (>500× mean coverage) [3]. For liquid biopsies, these tests are commonly run at far greater read depths (>5000× mean coverage) and require use of molecular barcodes to achieve acceptable sensitivity and specificity for samples with low (<5 %) tumor purity [5, 35].

Achieving high mean read depths with broader capture methods such as WES or WGS is costly and inefficient if the clinically reported regions are limited to known hotspots or a selection of cancer driver genes; therefore, WES and WGS are less suited to routine diagnostic applications. Additionally, achieving a sequencing library with sufficient molecular complexity (number of unique molecules) to drive a whole exome or genome target to >500× coverage is challenging, particularly from FFPE-derived materials. Many diagnostic services sequence tumor material only, without matched normal germline data from the same patient (for example, whole blood). Analytically, this approach is more tractable if the area being interrogated is smaller than a whole exome or genome.

However, in the immunotherapeutics field, WES might be a more appropriate test than a gene panel for the purposes of clinical management. Despite encouraging recent successes in immunotherapeutics (for example, the approval and use of checkpoint blockade inhibitors in a range of cancers), the understanding of predictors of response is incomplete [46]. Recent work has shown that mutational load and neoantigen load might be more useful biomarkers of response than specific driver gene mutations [47]. Similarly, the determination of mutational load and neoantigen expression is more predictive when whole exome data are used compared to large or small gene panels [48].

In cancer, WES is most commonly used in the clinical research setting, though diagnostic applications have been described [49]. One of the difficulties of WES for researchers is the so-called “long tail” of cancer genes, that is, the distribution of cancer-related genes with low frequencies in particular tumor types [50]. To address this phenomenon, research projects such as TCGA performed WES on a broad range of tumor types in an effort to better catalog the vast majority of these low prevalence cancer genes [2]. Recent efforts suggest that WES of liquid biopsies might be feasible to characterize metastatic and refractory tumors that would otherwise be challenging to biopsy [22, 51].

Single cell nucleic acid sequencing has been under development using many technologies. Single cell transcriptome profiling of tumor-derived cell populations is a highly sensitive and powerful tool for characterization of the tumor microenvironment and tumor heterogeneity [52]. Recent work by Tirosh et al. [21] highlights how this type of analysis could be leveraged in the future to profile tumors for likely development of drug resistance or candidacy for immune checkpoint blockade inhibitor treatment. Similarly, Miyamoto et al. [53] examined resistance development in prostate cancer using microfluidic enrichment of circulating tumor cells. Methods have been described for both RNA and DNA sequencing from single cells that leverage molecular biology techniques such as template-switching (Smart-seq) [54], incorporation of UMIs [55], and single nucleus sequencing [56]. Other methods have incorporated innovative technological platforms (nanodrops) to isolate cells and perform library construction at low cost, for example, Drop-seq [57] and the 10X genomics (Pleasanton, CA, USA) platform.

Bulk transcriptome sequencing and targeted RNA sequencing are now more widely adopted. Targeted RNA sequencing assays are used to capture and identify gene translocations in cancer samples [17]. Other sequence-based tests have been launched commercially that target common, potentially druggable oncogene fusions in *ALK*, *RET*, and *ROS1* in non-small cell lung cancer (NSCLC), a test historically carried out by immunohistochemical assays such as fluorescence in situ hybridization [58, 59]. Integrated analyses of exome (or genome) plus transcriptome profiles from a single tumor provide a more complete picture of the alteration landscape. Expression signatures from RNA can be used to determine if a driver gene candidate identified from DNA sequencing is actually expressed in the tumor or if resistance mutation expression levels change post-treatment [60].

## Sequencing technology

Just as selection of the “test” is dictated by intended use, the choice of sequencing technology (or platform) is also an important consideration. Although there is less dimensionality in the sequencing landscape today, with Illumina (San Diego, CA, USA) capturing most of the application space, the complexity, scale, cost, and required throughput of the test are important factors in determining the optimal platform.

The required read length and generation of paired end reads are a primary consideration. Read length is an important factor that relates to the type of genomic alteration events that might be queried and the overall accuracy of the placement of sequence reads relative to the target. In general, the most commonly used massively parallel sequencing platforms today generate short reads of a few hundred bases. This includes Illumina platforms (MiniSeq 2 × 150 bases, MiSeq 2 × 300 bases, NextSeq 2 × 150 bases, and HiSeq series 2 × 150 bases), also the Thermo (Waltham, MA, USA) Ion Torrent platform (Proton 1 × 200 bases), and the Qiagen (Hilden, Germany) GeneReader (100 bases). The utility of reads of this length is related to the type of assay being performed. For example, for amplicon sequencing (using “hotspot” panels), in general short read sequencing matches the size of the amplicon, and the amplicons can be designed such that the hotspot itself is located at a position where high quality can be expected (that is, not at the end of a read). Reads of a hundred or so bases are also useful for short variant detection using targeted sequencing of a gene panel or exome or in WGS. Similarly, for FFPE or cfDNA-derived materials, template lengths are generally shorter, so read lengths in the low hundreds of bases are appropriate.

Paired-end sequencing, which refers to sequencing a DNA fragment from both ends (the forward and reverse reads may or may not overlap), increases the utility of short reads in two ways. Some types of structural variation can be detected when the pairs of reads align to the genome in an unexpected way [61]. Sequencing both ends of fragments can also allow “de-duplication” in deep sequencing, where the occurrence of fragments with the exact same ends can be used to mask some reads as molecular duplicates, thus not adding to library complexity (for example, the MarkDuplicates tool in Picard [62]).

The main limitation of short reads (even if paired end) is in the discovery of fusion events or structural variation. Detection of known fusion events can be enabled by targeted assays that increase the utility of short reads by requiring mapping to a small or predefined event. Alternatively, specialized library construction methods to create long insert mate-paired libraries have shown some successes in structural variation detection [63]. For discovery of novel rearrangements, the most powerful approach involves long reads in which fusion or rearrangement events are spanned within the read. Options here include Pacific Bioscience (Menlo Park, CA, USA) instruments that generate reads of thousands of bases or the use of approaches such as the 10X Genomics platform, which links together short reads using a molecular barcoding approach. Another platform under active development in the long read space is the nanopore-based sequencing technology commercialized by Oxford Nanopore (Oxford, UK).

Ideally, the generation of very long reads would cost the same as an equal coverage of short reads, but this is not the case. Most dramatic decreases in sequencing cost have come from the platforms that generate short reads. For example, release of the Illumina HiSeqX decreased cost by threefold compared to the HiSeq2500: sequencing of a 30× human genome cost approximately $1500 on the HiSeqX compared to $5000 on the HiSeq2500. Sequencing the whole genome with long reads on a platform such as Pac Bio is cost prohibitive in most settings, at $20,000–80,000 per sample. In general, long read sequencing is used to sequence smaller (such as microbial) genomes or to target complex regions of the human genome (such as human leukocyte antigen genes) that are intractable for short read sequencing.

Short read sequencing costs vary considerably by platform, based on the instrument yield. For example, the lowest cost per Gb (billion bases) on a short read sequencer is approximately $15/Gb on the HiSeqX platform with an output of 1800 Gb bases per run. This level of throughput is appropriate for WGS which requires at least 100 Gb of data per sample, or considerably higher for tumor sequencing. Lower throughput platforms such as the MiSeq and HiSeq 2500 cost considerably more per Gb ($200/Gb and $45/Gb, respectively) but have an output per run (15 Gb for MiSeq, 1000–1500 Gb for HiSeq 2500) more appropriate for smaller scale sequencing, such as the panel test. A panel test of 100–200 genes might require 0.5–1 Gb per sample. Platform selection for this level of sequencing is a balancing act between the competing pressures of cost and turnaround time. To run most efficiently, multiple samples would be indexed, pooled, and sequenced on enough lanes to achieve the desired coverage. In practice, in the clinical testing world, the need for more rapid turnaround times necessitates running incomplete, and thus more expensive, batches. Technical features, such as template preparation techniques, sequencing chemistry, and error profiles are also important considerations. A review of technical differentiators is presented by Goodwin et al. [64].

## Analytical considerations

**Fig. 2:**
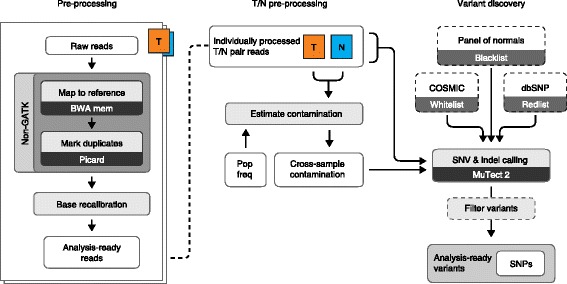
Example of a best practices SNV calling workflow for somatic exome and genome data (reproduced with permission from [80]). Raw reads from the sequencing instrument are aligned and duplicate reads are marked (using the Picard tool). Vendor-assigned base quality scores are recalibrated for accuracy (based on position in read and other factors). Before running somatic analysis, both tumor and normal read groups are assessed for contamination, such as sample swap, cross-contamination, and tumor contamination in the normal sample. Somatic variants are those passing filter variants that are present in the tumor but not in the matched-normal sample. Several filters are used to control for technical noise in the system, which includes the variant allele frequency and a panel of normals (for more details see Cibulskis et al. [45])

Identification of somatic mutations of different types requires individually optimized approaches. There are many commonly used somatic variant callers each with varying performance attributes and optimizations [65]. In our own group, we are moving toward local re-alignment-based approaches for calling point mutations, insertions, and deletions (that is, Mutect 2, which utilizes the Haplotype Caller module of GATK [66] to call both single-nucleotide variants and indels). Fig. 2 provides an example of a best practice somatic calling workflow using GATK-Mutect. Considerations for single-nucleotide polymorphisms and InDel calling include depth of coverage and base quality scores. Base quality scores are often recalibrated from instrument-provided scores to account for context-specific and systematic variation in a process known as base quality score recalibration (BQSR). Somatic variant calling for very low allele fraction events, such as those in cfDNA, requires additional components. For example, these methods often use UMIs to enable more precise de-duplication and error correction of amplified libraries [35].

Structural variation (such as duplication, copy number variation (CNV), inversions, and translocations) has traditionally been difficult to call with standard short read data. WGS is the most well characterized data type for structural variation calling, particularly when supplemented by long linking information or long reads. Recent technological advances that use droplet partitions (emulsions) and unique molecular barcodes have made this data type more tractable [67].

Some methods for variant calling rely on having a matched normal sample from the same patient to filter individual germline variants, which would otherwise be considered false-positive somatic calls. Additionally, a set of data created with non-cancer samples that uses the exact same assay and sequencing technology, a so-called “panel of normals” (PoN), is useful for removing artifacts due to systematic process variation in the library preparation or sequence generation steps [45]. Specific PoNs are needed for each different process type, for example, cfDNA low input library construction requires its own PoN for filtration. Some groups do not use matched normal material. In order to minimize false-positive calls, these groups either focus on calling previously characterized driver events in known oncogenes (in the case of hotspot panels), or use advanced filtering methods—unmatched normal, PoN, large germline databases (for example, 1000 Genomes, ExAc)—to remove non-somatic variants [48]. Specificity can be further increased by review of candidate mutations by an experienced molecular pathologist and cross-referencing somatic mutation databases such as COSMIC for pathogenicity information [48].

An area of particular interest at present is immunoinformatics, which refers to the analysis of patient genomics data to profile their immune system, and in the case of cancer patients, the tumor microenvironment, with the aim of identifying biomarkers of response to immune blockade inhibitors [47]. Software tools now exist that use patient exome and transcriptome data to call HLA types and predict T- and B-cell epitopes. For a review of these methods, see Backert and Kohlbacher [68]. T-cell receptor (TCR) profiling through targeted amplification and sequencing of the CDR3 region is another application that has seen adoption for both diagnostics [69] and clinical research [70].

Accurate analysis of CTC single-cell data is confounded by the errors imparted by the WGA process. WGA introduces allelic distortion and polymerase errors that result in exceedingly high false-negative and false-positive rates, in contrast to bulk sequencing, and affect our ability to confidently detect all classes of genomic alterations [34]. Strategies to overcome the error modes of WGA include joint analysis together with bulk sequencing of matched tumor tissue or other independently amplified single cells [22, 71]. These methods are reviewed by Gawad et al. [72].

So far, we have discussed only the technical aspects of analysis to identify somatic variation in the patient’s tumor. Depending on the size of the territory interrogated, the number of somatic variants found can range from a few (in a hotspot panel) to a few hundred (in a whole exome). The next step in the process prior to clinical decision-making is the annotation of variants with functional information and interpretation of the likely impact of the events in the context of the patient’s disease. For germline diseases, molecular geneticists routinely use large population variant frequency databases, such as ExAc [73], to filter out events previously found in the population. These same resources can be used to filter germline events from somatic variation [48] but are not useful for annotation or filtration of actual somatic events. To annotate and filter somatic events, a large database of somatic variation, COSMIC, is often used [74] and, increasingly more clinically curated databases such as ClinVar [75] are used to query the pathogenicity of specific variants. Unfortunately, a lot of deep knowledge about specific tumor type variation still resides in proprietary databases maintained by commercial diagnostic companies, though efforts are underway to free or recreate these datasets and others as publically available resources [76–78]. Finally, given the complexity of the data types and the number of variables that can impact the results, there is still a need for expert human review in the field of clinical genomics. Typical activities for molecular geneticists, pathologists, and in some cases molecular tumor boards (comprising specialists who discuss the results of advanced genomic diagnostic tests of cancer patients), range from variant review and visualization, using tools such as the Integrated Genome Viewer (IGV) [79], to prioritization of variants based on clinical or professional experience and the context of the patient’s disease.

## Conclusions

Never before in the history of molecular oncologic pathology have we had the ability to examine a patient’s tumor with the resolution or richness of information that it is possible to generate today. With this increased resolution comes a lot of additional considerations. In order for genomic information to be useful in a clinical setting we need the data produced to be accurate, actionable, and timely. Advances in sequencing technologies have made the sequence data itself extremely accurate in most contexts, such that the major sources of false positives and false negatives today are caused by pre-analytical factors (such as chemical or physical damage of DNA/RNA, limited material, or inappropriate handling) and post-analytical factors such as variant calling limitations. Upfront consideration of intended use of genomic data and careful selection of both assay type (exome, transcriptome, targeted panel) and bioinformatic analysis methodology are required for optimal utility. Future advances in solid tumor clinical research will likely see more integrated analyses of a tumor. That is, not just a targeted gene panel test, but a targeted panel, plus a targeted fusion test, plus an immune cell profile. A more expansive profiling, which offers the ability to cross-validate findings and gain a more complete molecular picture of a tumor, could incorporate a deep whole genome (with linked reads for SV detection) plus a transcriptome (for expression, fusions, and variant validation) plus an epigenetic test (for dysregulation). The methods for such testing exist today but require continued optimization to work with available sample types and amounts and more integrated analytical platforms to bring the multi-omic datasets together in a meaningful and practically interpretable way.

Liquid biopsy represents an exciting new class of sample matrix that enables more frequent and facile monitoring of tumor burden and could allow for more rapid treatment course correction. Further advances in liquid biopsy methodology could enable not just post-diagnostic sampling but also pre-diagnostic screening for cancer risk, as has been shown with the application of cfDNA in the non-invasive prenatal testing (NIPT) field. With continued technological advances and increasing availability of variant databases for annotation and interpretation, the use of genomic testing in clinical cancer management seems likely to continue to progress toward standard of care, though non-trivial issues such as access to testing, wide-spread physician education, and adoption of testing, and reimbursement for testing will likely be the rate limiting steps.

## Abbreviations

AF: Allele fraction
cfDNA: Cell-free DNA
CNV: Copy number variation
CTC: Circulating tumor cell
ctDNA: Circulating tumor DNA
EV: Extracellular vesicle
FFPE: Formalin-fixed paraffin-embedded
NIPT: Non-invasive prenatal testing
PBMC: Peripheral blood mononuclear cell
SNP: Single-nucleotide polymorphism
SNV: Single-nucleotide variants
SV: Structural variation
TCGA: The Cancer Genome Atlas
UMI: Unique molecular index
WES: Whole exome sequencing
WGA: Whole genome amplification
WGS: Whole genome sequencing
WTA: Whole transcriptome amplification
